# Situating emotion regulation in autism and ADHD through neurodivergent adolescents’ perspectives

**DOI:** 10.1038/s41598-025-21208-x

**Published:** 2025-10-27

**Authors:** Georgia Pavlopoulou, Susie Chandler, Steve Lukito, Myrofora Kakoulidou, Maciej Matejko, Isabel Jackson, Beta Balwani, Tiegan Boyens, Dorian Poulton, Luke Harvey-Nguyen, Zoë Glen, Archie Wilson, Elisa Ly, Elizabeth Macauley, Jane Hurry, Sylvan Baker, Edmund J. S. Sonuga-Barke, The RE-STAR team, Georgia Pavlopoulou, Susie Chandler, Steve Lukito, Myrofora Kakoulidou, Jane Hurry, Sylvan Baker, Edmund J. S. Sonuga-Barke, Andrea Danese, Johnny Downs, Eloise Funnell, Kirsty Griffiths, Lauren Low, Umaya Prasad, Angus Roberts, Emily Simonoff, Daniel Stahl, Anna Wyatt, Graham Moore, Dennis Ougrin, Amanda Roestorf, Rebecca Kirkbride, Claire Lewis

**Affiliations:** 1 Division of Psychology & Language Sciences, Faculty of Brain Sciences, Group for Research in Relationships And Neurodiversity (GRRAND), Research in Clinical, Educational & Health Psychology, University College London, London, UK; 2 Anna Freud National Centre for Children and Families, London, UK; 3 School of Academic Psychiatry, Institute of Psychiatry, Psychology & Neuroscience, King’s College London, London, UK; 4 University Hospitals of Leicester NHS Trust, Leicester, UK; 5 UCL Institute of Education, London, UK; 6 Royal Central School of Speech & Drama, London, UK; 7 Cardiff University, Cardiff, UK; 8 Queen Mary University of London, London, UK; 9 Autistica, London, UK; 10 Place2Be, London, UK; 11 ADHD Foundation, Liverpool, UK

**Keywords:** Autism, ADHD, Resilience, Emotion regulation, School mental health, Human behaviour, Translational research

## Abstract

Accounts of emotional dysregulation in autism and ADHD are typically based on external adult observations anchored in neurotypical notions of appropriate emotional responses1. These often fail to account for neurodivergent people’s efforts to regulate in the context of the upsetting experiences they face, and to explore how neurodivergent young people manage emotional burden or find effective strategies to reduce distress and increase resilience. We interviewed 57 adolescents (11-15 years; 19 females) with diagnoses of ADHD (n=24), autism (n=21) or both (n=12), about their experience of upsetting events using a codesigned semi-structured interview schedule. We analysed data using reflexive thematic analysis which generated shared themes with diagnostic nuances. Three themes were found: (1) What helps prevent experiences from becoming upsetting, (2) Managing emotional responses during periods of upset and (3) Leveraging own strengths. Participants highlighted the importance of consistent, neurodivergent-affirming environments, flexible supports, and being genuinely accepted. Autonomy in choosing self-regulation strategies, co-regulation and expressing distress without stigma were key to emotional wellbeing. These findings underscore the value of stable, trusting relationships and the need for predictable routines. Results offer actionable insights for educators, caregivers, and professionals aiming to foster inclusive, emotionally supportive environments for neurodivergent youth.

**Supplementary Information:**

The online version contains supplementary material available at 10.1038/s41598-025-21208-x.

## Introduction

Adolescence is a pivotal period for the emergence of mental health difficulties. Neurodivergent young people - those with diagnoses such as autism and attention deficit/hyperactivity disorder (ADHD) - are disproportionately affected, being significantly more likely to experience depression, anxiety, and suicidal ideation^1^. Much of this distress is linked to school experiences, where rigid behavioural expectations, sensory overload, and social exclusion render daily life overwhelming. Research highlights these factors can contribute to school absenteeism, underscoring the importance of support in educational settings^2,3^.

A recent National Autistic Society^4^ survey found that only 26% of autistic students report being happy at school. The Office for National statistics similarly reports that the majority of autistic and ADHD students struggle with peer relationships, with those lacking friendships often feeling like “outsiders” and finding break times “depressing”^5^. Despite these figures, mainstream school-based mental health services are rarely adapted to support neurodivergent needs-contributing to academic disengagement, exclusion, and elevated risks of long-term mental health issues or even premature mortality^6^.

A key contributor to these outcomes is emotion regulation difficulty - often manifesting as irritability, reactivity, or mood instability - in both ADHD^7–12^ and autism^13–18^. However, dominant conceptualisations of emotion regulation are grounded in neurotypical norms, casting neurodivergent emotional expression as disordered or deficient. Often overlooking young people’s own insights and lived experiences, this risks pathologizing emotional difference.

Because most tools used to assess and address emotion dysregulation in neurodivergent populations are based on neurotypical emotional frameworks, the distinct ways in which autistic and ADHD individuals feel, process, and express emotion are often misunderstood or labelled as dysfunction. Widely used interventions like PATHS^19^ and Zones of Regulation^20^ exemplify this deficit-based model. Even well-intentioned adaptations, such as EASE for autism^21^ and RELAX for ADHD^22^, generally maintain the assumption that effective emotion regulation requires conforming to neurotypical standards through cognitive control. Yet evidence increasingly shows these approaches to be limited in effectiveness for neurodivergent youth^21,23,24^. They often ignore the sensory, social, and relational burdens that drive distress and may encourage masking behaviours, which can lead to further psychological harm^23^. Crucially, they are not neurodiversity-affirming, often devaluing atypical emotional expressions that may be adaptive or communicative, while overlooking the expertise neurodivergent youth have in managing their emotions.

In the Regulating Emotions – Strengthening Adolescent Resilience (RE-STAR) research programme, we take a different approach to understanding emotional responding, informed by qualitative interviews with 57 autistic and ADHD young people and shaped by their participation as co-investigators^25^. Pavlopoulou et al.^26^ identified four recurring themes evoking what is often labelled “emotional dysregulation”: (1) social dislocation and conflict, (2) masking, (3) self-doubt and shame, and (4) sensory mismatch. While themes were broadly shared, the content diverged: ADHD youth often described being triggered by unjust or controlling actions from others; autistic youth more frequently cited alienation and a sense of not belonging. Masking also differed - autistic individuals tended to hide negative emotion to fit in, while ADHD youth masked to avoid punishment.

These findings support a shift in interpretation. Many mental health challenges traditionally attributed to internal deficits in emotion regulation may instead stem from high levels of emotional burden carried by neurodivergent individuals as they seek to manage everyday events. Neurodivergent youth face roughly twice as many upsetting experiences and respond with roughly double the emotional intensity compared to neurotypical peers^27^.

This paper returns to the qualitative interviews reported in^26^ to explore how neurodivergent young people manage these emotional burdens, with the goal of identifying strategies that reduce distress and promote resilience. In line with recent scholarship on inclusive research practices^25,28,29^, the study team made a conscious effort to embed participatory design at every stage. This involved actively involving neurodivergent adolescents in shaping the research focus, materials, and analytic process, with the aim of reducing power imbalances and ensuring that knowledge production was both accessible and meaningful to participants.

## Methods

### Ethics

The study was approved by the Health and Social Care Research Ethics Committee A (HSC REC A; reference 22/NI/0017). The research was performed in accordance with relevant guidelines/regulations, including the Declaration of Helsinki. Informed written parental/carer consent and young person assent were obtained for all participants.

### Coproduction with RE-STAR’s youth researcher panel

**Fig. 1:**
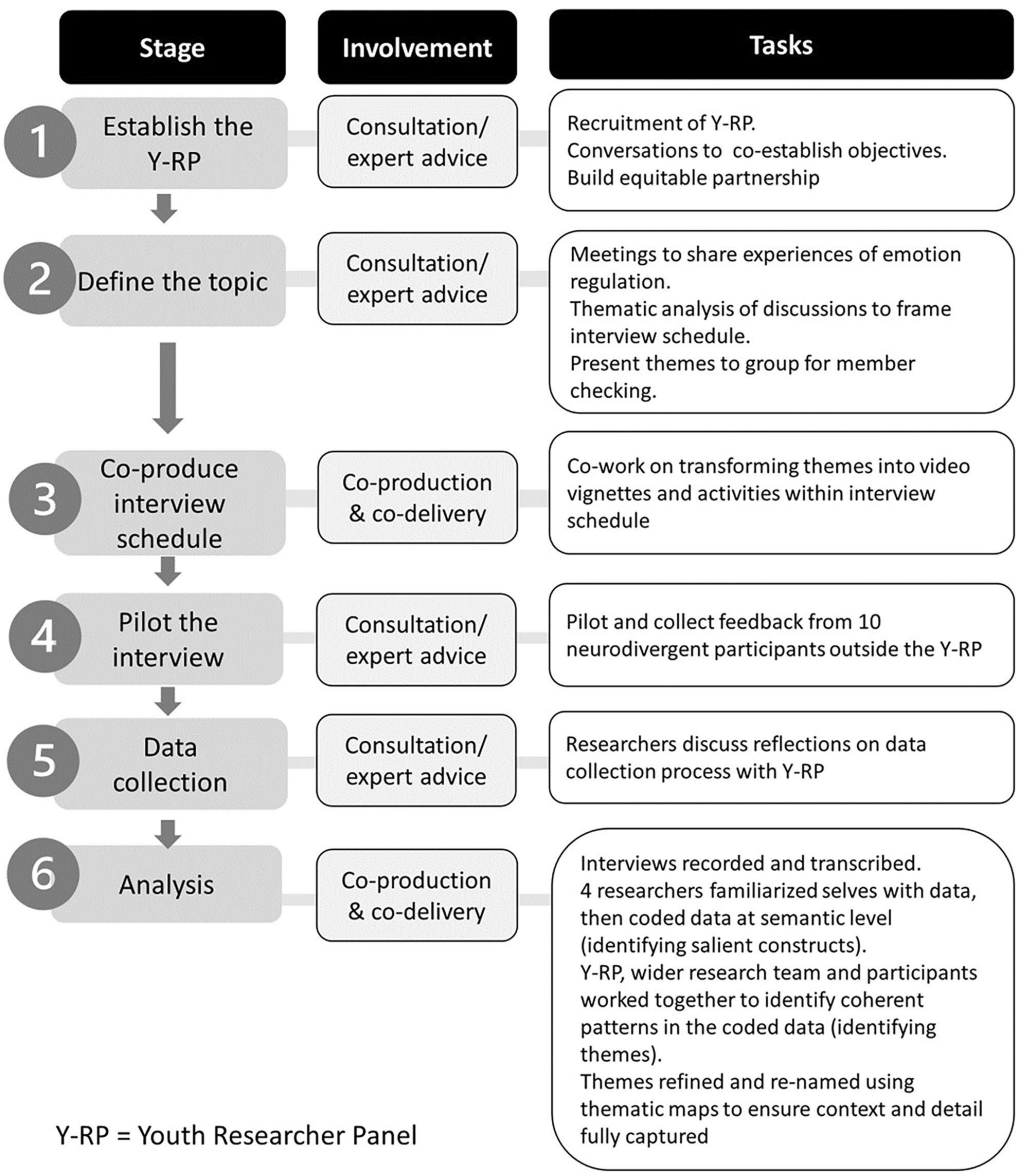
Stages of co-production.

The current study was co-designed with 10 young people between the ages of 18 and 25 years with diagnoses of ADHD and/or autism (Youth Researcher Panel (Y-RP)^25^ who were reimbursed for their time^30^. The collaboration between academic and youth collaborators was informed by an experience-sensitive approach^31,32^ according to which youth co-researchers are supported to contribute more fully to the research process, shaping its purpose and direction. Figure 1 illustrates the implementation of this approach.

### Participants

Fifty-seven young people aged 11–15 years participated in the interviews. The inclusion criteria were: (i) enrolment in a mainstream secondary school in the UK, (ii) sufficient English to participate in the interviews, and (iii) a formal diagnosis of autism, ADHD, or both from an NHS provider. They were recruited via local National Health Service (NHS) clinics and national charities’ networks via newsletters/social media. Participants were assigned to the following groups based on their validated clinical diagnosis: ADHD (n=24); Autism (n=21); ADHD+Autism (n=12), hereafter referred to as ‘dual diagnosis’. Participants did not report cooccurring mental health diagnoses. The sample was 67% male, and 75% white. 16% received free school meals. Eligibility for free school meals was recorded as a proxy indicator of socioeconomic status, to provide contextual information about participants’ background (see Table 1 for sample characteristics).

**Table 1:** Sample characteristics.

	ADHD (n=24)	Autism (n=21)	ADHD+Autism (n=12)
Mean age in years (SD)	13.0 (1.40)	12.9 (1.35)	13.2 (1.4)
Sex N (%):			
Male	18 (75.0)	11 (52.4)	9 (75.0)
Female	6 (25.0)	10 (47.6)	3 (25.0)
Receiving free school meals N (%)	5 (20.8)	2 (9.5)	2 (16.7)
Ethnicity N (%):			
Asian/Asian British	1 (4.2)	0 (0.0)	2 (16.7)
Black/Black British/Caribbean/African	1 (4.2)	1 (4.8)	2 (16.7)
Mixed/multiple ethnic groups	2 (8.3)	3 (14.3)	-
White	19 (79.1)	16 (76.2)	8 (66.7)
Other	1 (4.2)	1 (4.8)	-

### Materials

A qualitative interview schedule was co-developed with the Y-RP. They contributed to the question route, created video ‘vignettes’ to provide interviewees with examples of common upsetting events (to prompt them to talk about their own experience), and piloted a ‘creative task’. The ‘creative task’ was designed to encourage participants to think in different ways about emotional experiences and to provide an opportunity to discuss issues beyond a traditional question and answer approach which can encourage reliance on repetition of existing discourses on certain topics. See Supplementary Material I for the ‘Creative task’ prompts and examples, and interview prompts. The interview schedule was piloted with 10 neurodivergent participants after which minor changes were made to the procedures. No changes were made to the interview schedule itself.

### Procedure

Parent/carers of potential participants completed a brief online screening questionnaire to confirm eligibility for the study. ‘Eligible’ parent/carers were sent an information letter, consent/assent forms, and a visual guide on the study process. Parent/carers were asked whether their child was aware of their autism/ADHD diagnosis, the degree to which they understood and identified with their diagnosis, and what terms they preferred to be used when speaking about autism/ADHD.

#### Orientation session

This lasted 15 to 30 minutes, depending on participant availability. Parent/carers and adolescents met with the researcher via Zoom. Participants were introduced to the goals of the study and the idea of using creative tasks to illustrate upsetting experiences and related emotions. Participants were given a warm-up activity and walked through the interview schedule. They were then invited to share their ideas around the topic and choose a medium (drawing, photos, poetry) for the creative task. Participants were then given two weeks to complete the creative task prior to the interview session.

#### Main interview session

The interviews took place on Zoom and lasted 45 to 90 minutes. Parents were not present during the interviews, but they were nearby and available if needed.

The interview began with participants presenting their creative work. An adapted version of the ShowEd protocol^33–35^ was used to discuss the meaning of this work. Participants then answered questions while taking part in interactive activities such as picking vignettes which probed participants’ ideas and feelings about experiences they found emotionally challenging. The interview was used to examine how participants understood the nature of emotional provocations/triggers of and how they managed these. All interviews were audio- and/or video-recorded, with appropriate written parental consent and young person assent, and formally transcribed verbatim. To facilitate discussion, participants completed a creative task at home prior to the interview, where they produced an artwork, photo, or object to represent moments that felt calming, reassuring, or upsetting. See examples in Supplementary Material II.

During the interview, young people were invited to describe these tasks (e.g., *“What is it? How does this relate to your feelings? What situations does it remind you of?”*). In addition, participants engaged with short vignette scenarios presented on screen (e.g., a classroom conflict with a peer, being blamed unfairly, or managing group work). They were asked to reflect on whether the situations felt familiar and how they might respond emotionally, for instance: *“What’s your emotional reaction in a similar situation?”* and *“What helps you to prevent negative emotion?”*.

At the end of each session, young people were debriefed in age-appropriate language, given the opportunity to ask questions, and provided with information on available support resources.

### Analysis

Interview transcripts were analysed line-by-line using inductive, reflexive thematic analysis, following^36,37^ and^38^. first 10 interviews were coded by 4 researchers, 2 of them neurodivergent, to standardise coding practices. The rest of the interviews were split across two interviewers who independently coded them. Two Y-RPers also coded a sample of transcripts of participants matching their diagnostic group membership. Next, the initial findings were presented to the team of academic researchers and Y-RPers who gave feedback on emerging codes and their clustering. Codes were clustered into initial themes during group discussions between the academic researchers and Y-RPers. The results were not structured according to the research questions, but rather to themes that appeared across the interviews as a whole. While all themes were relevant to neurodivergent young people regardless of their diagnosis, at times the interpretation and content of subthemes differed between the three groups. The team noted contrasts within each theme. A final set of themes was agreed through an iterative analytic process of multiple sessions between the academic researchers and Y-RPers. Codes were discussed with a subsample of study participants (n=6), who provided feedback on the near-final themes, written in lay language. Final themes were discussed and refined, in both one-to-one and group meetings, by an interdisciplinary and neurodivergent team of psychiatrists and psychologists, both clinical and non-clinical.

### Positionality and reflexivity

The subjectivity of the academic researchers and the Y-RP was explicitly discussed during weekly and monthly meetings, recognising the group’s familiarity with the research topic on both a scientific and personal level. This included both the different types of expertise and experience individuals brought to the research, as well researchers’ and Y-RP experience of neurodivergence. According to^39^, such an approach is superior to the use of ‘unknowledgeable’ coders, who may be less able to add richness that ‘insider’ researchers often bring to the coding process.

## Results

Through visual artwork, verbatims, and short commentaries, participants shared how they understood and managed upsetting emotion. Their insights revealed strategies used to prevent distress, regulate emotions, and draw on personal strengths. We identified three overarching themes:

1. What helps prevent experiences from becoming upsetting.
2. Managing emotional responses during periods of upset.
3. Leveraging own strengths.

We explore these themes and their diagnostic nuances below.

## Theme 1. What helps *prevent* experiences from becoming upsetting

**Fig. 2:**
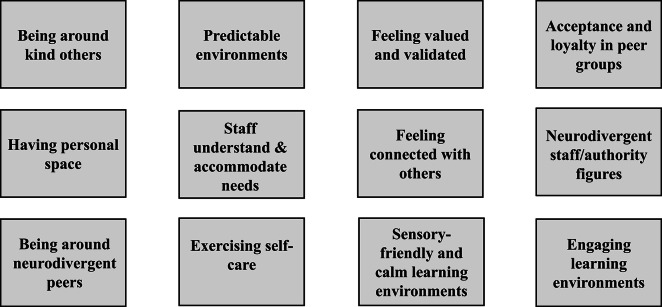
Latent codes for what helps prevent experiences from becoming upsetting.

The latent codes this theme are presented in Figure 2 below and described in more detail below.

### Connections and acceptance over rules and expectations to fit in

All young people highlighted the positive impact of “someone being kind” (male, 13, autism) in helping them stay grounded and respond more calmly to difficult situations. Participants most often described such experiences in school settings, though several also emphasised the importance of supportive relationships at home. They explained they could manage their emotions for longer when people would *“just be kind when asking me something”* (male, 11, dual diagnosis), and when they felt *“accepted”* (female, 13, ADHD) as their authentic selves - especially outside the home. This sense of belonging was most often found during regular, predictable time with trusted people—such as “*time with my friends*” (male, 15, dual diagnosis) or “*playing family games*” (male, 13, dual diagnosis). These relationships helped buffer against school-related stress and prevented emotional overload.

ADHD participants, in particular, stressed the importance of being accepted by peers without being judged:

“*Some people say I’m weird because of how I can laugh at anything…or they also think I’m weird because of how I can’t stop moving… I’m weird, okay but why? You know me… You know I’m like this…”* (male, 12, ADHD)

For autistic participants, emotional challenges were experienced less intensely when they could spend time with people who shared “*shared interests or similar profiles*” (female, 15, autism):

“*The student body being bigger and relating to me a lot more is really, really helpful and inspiring… We share a lot more in common… and just generally from my experience with them, they’re all very nice people… They’re what I need in this stage of life.”* (female, 15, autism)

ADHD participants often spoke about loyalty, both receiving and showing it. One young person said they felt good when they could “defend friends” (male, 13, ADHD). Others valued being quietly supported or simply allowed to exist in their own way:

“*…Some of my friends will help the best they can. Some of them will just let me do my own thing, which I do appreciate…”* (male, 13, ADHD)

Autistic participants highlighted the importance of environments where differences in how people think are not only accepted but valued. They felt this could help challenge damaging stereotypes:

*“Using as an insult or calling somebody autistic… it should be taught more in schools… because people have no idea what it is and the impact…”* (female, 12, autism)

All participants, particularly those with a dual diagnosis, said they coped better when supported by people who understood their needs. Helpful accommodations included: fidget toys, flexible seating, less attention on mistakes, personal space, and clear explanations. Engaging lessons also made a difference:

“*If the subject’s like good or fun, then I will be like, zoned in… Something happens in a subject which I find exciting and yeah*!” (male, 13, dual diagnosis)

“*Really calm lessons. Maybe they’re a bit easy. A bit relaxed. Not too hard or strict*.” (male, 14, dual diagnosis)

These examples illustrate how classroom environments and teaching style at school played a direct role in shaping emotional wellbeing.

Some felt that being understood could reduce unnecessary discipline-related stress:

“*They could know the fact that I do have ADHD… And then friends, including teachers… can start to give me more chances and opportunities to fix that thing, instead of telling me off straight away.”* (male, 13, dual diagnosis)

For certain autistic participants, connecting with neurodivergent staff or peers was especially powerful:

“*What’s been helpful for me at school is having teachers who are neurodivergent… She understands what I go through a lot more… She understands how I’m feeling, it just works.”* (male, 15, autism)

“*They know that [change] can be stressful… If they tell us in advance… not in a very condescending way… It’s not like a super-duper big deal for me. I don’t know*.” (female, 15, autism)

“*There’s a place I can go called XXX… it’s full of a bunch of other kids, like me… So it just makes me feel not out of place*.” (female, 11, autism)

### Self-care as protective practice

Autistic participants, whether or not they also had ADHD, frequently described self-care as a foundational way to protect their emotional wellbeing and minimise the risk of becoming overwhelmed. These young people framed self-care not as a reactive measure, but as something purposeful and preventative—woven into their daily routines and deeply aligned with their sensory and cognitive needs. While many of these practices were performed at home, they were often described as protective in managing the demands of school.

Participants consistently emphasised the value of engaging in activities that were predictable, low-pressure, and personally meaningful that created calm, restored energy, or brought gentle joy. Crucially, these practices were seen as effective precisely because they did not require high effort or emotional explanation. As one participant shared, it helped to do “*something that calms me down… something at a slower pace*.” (male, 12, autism)

Sleep and rest emerged as recurring themes:

“*I just try to get to sleep. I’m trying to calm myself down from what was going on*.” (male, 12, autism)

Others mentioned quiet or solitary routines as a way to recentre and stay well:

“*I usually just meditate and read to just, like, clear my mind*.” (female, 15, autism)

“*I listen to music and then most of the time I’m fine.”* (female, 15, autism)

“*Sometimes I just want it to be like, quiet, so I can just think of my own thoughts rather than just thinking of all the people talking and overhearing what they’re saying or anything… I would probably -- I would prefer to be quiet because then I can just have my own like thoughts and be able to like hear the people I’m actually going to be talking to.*” (female, 12, autism)

In these examples, participants drew attention to the sensory relief and mental space created through stillness and having space alone. For some, this involved small sensory resets:

“*Drinking water usually helps me, because I have something else to focus on*.” (male, 14, autism)

Others found gentle distraction through familiar digital media:

“*Or I can distract myself with watching funny videos on YouTube on my laptop or on my phone or playing something fun that can distract me from my stress.”* (female, 12, autism)

Creative expression also played a restorative role, offering participants a way to reconnect with a sense of self-worth:

“*I think writing helps me a lot because it helps calm me and realise that I am useful at loads of stuff. And I think that really helps the also, like, general distractions like reading.”* (male, 15, autism)

Some of the most vivid self-care descriptions came from participants who engaged with the natural world. One autistic young person described a recurring ritual of visiting a flower bush near his home:

“*There have been many times, I’ve brought flowers home as gifts, because that day I went to the little flower bush to chill. Each season brings a different flower, on spring, there’s a high abundance of lavender where there’s lots and lots of lavender flowers. So I usually bring some back. In fact, outside of my house right now, there’s a little bush of lavender flowers…*” (male, 12, autism)

These examples highlighted how self-care was rooted in sensory regulation, meaningful distraction, and emotional grounding. Participants not only recognised what worked for them but also spoke of these routines as essential to navigating daily demands—particularly in environments that felt unpredictable, overstimulating, or socially challenging. In this way, self-care was not simply about managing emotional fallout but actively safeguarding wellbeing in advance.

## Theme 2. Managing emotional responses ‘during’ periods of upset

**Fig. 3:**
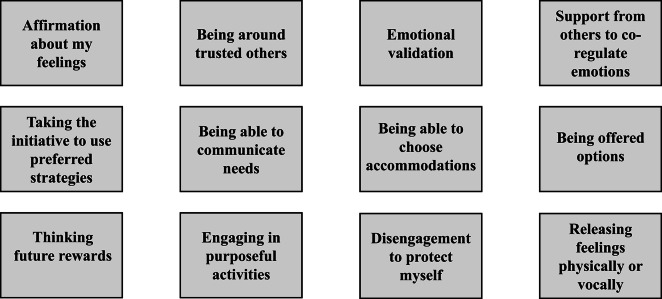
Latent codes for what helps manage emotional responses during periods of upset.

The latent codes for this theme are presented in Fig. 3, and described in more detail below.

### Affirmation by and “check-ins” from others

When experiencing emotional distress, autistic participants placed strong emphasis on the importance of being seen, heard, and accepted. Many described how critical it was for others , especially trusted peers or adults, to acknowledge their feelings without judgment or dismissal.

“*It’s nice when they listen to me because they’re like they want me to feel good.”* (female, 13, autism)

“*They ask me if everything is okay and that kind of thing. It makes me feel better when they do that. Because they’ve acknowledged it. Just ask me what’s wrong.”* (female, 13, autism)

“*I’d like the person to accept me and accept my emotions*.” (male, 13, autism)

This kind of emotional validation - being recognised rather than corrected - was a recurring need. Many participants spoke about the emotional relief that comes when people check in on them, even if they’re not openly expressing distress.

*“They ask me what’s wrong, and they acknowledge my existence.”* (male, 13, autism)

“*I wish they knew that when I’m upset, I don’t tell anyone. I just like, turn off. I wish they knew that and were able to notice when there’s something wrong, but that’s quite a hard thing to do.”* (female, 13, autism)

For participants with autism or a dual diagnosis, affirmation wasn’t just comforting—it could also be emotionally regulating, helping them gain clarity or restore calm. Reassurance, predictability, and gentle emotional prompts were identified as particularly helpful:

“*If I ask and she tells me what’s going to happen, then that stops the stress entirely. Or if I find out what’s gonna happen maybe the next day*.” (male, 13, dual diagnosis)

“*It usually takes someone telling me that I’m not what I think I am to make myself feel better and then once I’m out of that state if I start telling myself that I am alright then my mood will start increasing again*.” (male, 13, dual diagnosis)

Some participants described feeling emotionally “stuck”—not knowing how to shift or soothe their feelings on their own. In these moments, external support became especially important.

“*It’s nice when they listen to me because they’re like they want me to feel good.”* (female, 13, autism)

“*When with my friends, they’re just, I’m not complaining to them about something, so they just let it pass and try to cheer me up*.” (female, 12, autism)

Others explained that when overwhelmed—feeling “overheated” or emotionally flooded—decision-making becomes difficult, and they value being gently guided:

“*They clarified that choices and ideas stemming from self can be hard when feeling ‘overheated*.’” (male, 13, autism)

“*I would usually tell them [i.e., friends] that I don’t know what to choose. It’s making me feel weird and ask them to help me choose*.” (male, 12, autism)

“*They say, everything’s gonna be okay to fine. Nothing to worry about*.” (male, 11, autism)

These examples highlighted that in moments of emotional difficulty, what matters most was not advice or solutions, but attuned, non-intrusive presence, reassurance and affirmation that the young person was valid for feeling as they did. This was especially emphasised in school, where comments from teachers and peers was contrasted with the comfort of trusted support at home.

### Self-directed regulation: Autonomy and action during distress

For many participants—especially those with ADHD or a dual diagnosis—taking initiative played a central role in managing distress. These young people expressed a clear preference for self-driven strategies to de-escalate their emotions, solve interpersonal issues, and reshape their environment in ways that supported their wellbeing. They valued having a say, both in how they are supported and in how they navigate difficult emotions.

Some participants described how being able to communicate their needs, reflect on their behaviour, or propose solutions helped reduce the impact of distressing situations. This was particularly true when they had the freedom to experiment with self-suggested accommodations in the classroom. In school, these strategies often involved negotiating the physical or social environment, while at home participants described drawing on solitary activities or sensory regulation.

“*If I had a different seat then I would have attended those classes.”*

(male, 15, dual diagnosis)

They expressed a strong preference for being given options, rather than “*told to do things*” (male, 15, ADHD), and emphasized the need to feel they are “*treated like a person*.” (male, 13, dual diagnosis).

All participants with ADHD, including those with a dual diagnosis, emphasized the value of being allowed to take independent action in response to distress. This included removing themselves from triggers, problem-solving, and employing internal coping strategies such as positive self-talk, distraction, or thinking about future rewards.

“*Yeah, when I’m at school, I calm down by just kind of -- maybe I play a quick game on my laptop. We have our own laptops, so I’ll just play a quick game on my laptop and then get on with the work. Just quickly do something that’s different and then get on with the work*.” (female, 15, ADHD)

“*It’s like, if I’m upset then they’ll just ignore it, which I’ve told them that I feel comfortable with them ignoring it rather than talking to me about it.*” (female, 12, ADHD)

“*Probably they’ll just leave me alone*.” (male, 12, ADHD)

Some ADHD participants were also uplifted by purposeful engagement with personal values, especially kindness and altruism. These participants described moments of actively seeking hope and inspiration:

“*I’ll go online and I’ll look just like, basically good Samaritans, people being nice, people being incredibly generous. It is incredibly uplifting. It is an incredible mood lifter. I will like, I’ll be, I could be feeling like absolute crap. And I just watch a few of these videos, my mood is lifted completely out of the gutter... he’ll just go around being super generous to people. Just like giving them money to pay for hospital bills, stuff like that. It’s kind of like that happiness transfers onto me… seeing actual, like, pure joy from these people who have just like had, like, best moment of their life, it has made me cry on several occasions because it’s just incredibly uplifting to see*.” (male, 14, ADHD)

For autistic participants, self-directed action often involved gently disengaging from upsetting situations, taking time to reset through sensory soothing, kindness to self, or temporary withdrawal.

“*Being kind to myself.”* (female, 13, autism)

Participants described comforting routines such as having something to eat, taking a nap or shower, meditating, or praying. A common theme was the importance of being able to leave a distressing interaction or space.

“*I might just tell them to not talk to me. Do not talk to me for a while, while I sort out what’s going on*.” (male, 12, autism)

“*Walking away but not getting involved anymore. Because if I get involved anymore, when I’m upset, I’ll just get angry. And then it would go too far*.” (male, 13, autism)

“*Maybe going on a walk because I’ve noticed that fresh air is very good for me and so, I think that’s probably the main thing*.” (female, 12, autism)

### Letting it out: Physical and emotional release

In moments of intense emotion, participants described the short-term benefit of releasing their feelings physically or vocally. This form of expression—whether through crying, stimming, or yelling into a pillow—provided relief, even if it didn’t fully solve the underlying issue.

“*Crying. It just comes out and then, like, it might make me feel a bit better. Just get it out*.” (female, 13, autism)

“*I do stimming, and it helps me actually relax*.” (male, 11, autism)

“*I scream into my pillow. I scream into my pillow like this. I don’t know. I just, I like getting it off my chest. Oh, no, I don’t really achieve anything by being angry. When I rant, I feel like I’ve maybe got something off my chest.*” (male, 11, autism)

They also described a range of physical gestures used to communicate their emotional state—especially when verbalising was too difficult in the moment.

“*It was Friday and I had a substitute for science and then I got angry and I stormed off…I got angry…and walked out of the classroom.*”

(male, 12, ADHD)

“*I would stamp up the stairs to show her that I was angry at her… I’ll probably tut or huff or something… I’ll, not throw my phone, but I’ll put it down quite angrily to show that I’m angry… I’ll turn off my phone quite aggressively*.” (female, 12, ADHD)

## Theme 3. Leveraging own strengths - drawing strength from “within”

**Fig. 4:**
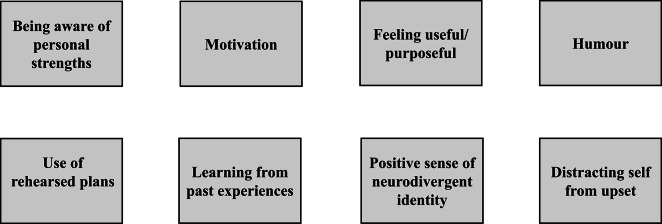
Latent codes for leveraging own strengths - drawing strength from “within”.

The latent codes for this theme are presented in Figure 4, and described in more detail below.

ADHD participants described being more able to both prevent and manage upsetting emotions when they recognised and actively engaged their personal strengths. These young people often relied on their own talents or activities that gave them a sense of achievement to help manage emotional distress. Some held this awareness independently, while others benefited from consistent praise and recognition by others, which helped reinforce their strengths. Most ADHD participants expressed motivation to succeed at school. For many, ambitions such as winning something or being the best at a skill acted as powerful protectors against spirals of negative thinking. Feeling useful and knowing they had contributed to something meaningful also supported their emotional resilience. These strengths were drawn on both at home (e.g., hobbies, creative activities) and at school (e.g., striving to succeed in class or sports). This theme illustrates how young people actively drew on their own resources and identity-protective strategies to maintain emotional balance. In particular, leveraging strengths provided a means of preserving autonomy, competence, and relatedness.

“*Then when I go out and help, I have actually quite fun. And I feel nice*” (female, 12, ADHD)

“*When you get an achievement for something like swimming or another sport…create something that is good, whether it’s Lego or something else*.” (male, 11, ADHD)

Participants with ADHD highlighted how specific traits, such as having a sense of humour or being distractible, could also be helpful in emotional regulation.

“*Let it go*” (male, 14, ADHD)

“*I get distracted really easily. So that helps take my mind off of what just happened.” (male, 13, ADHD)*

“*I can kind of coach my mind to just fight the boredom...”* (male, 13, ADHD)

Some explained that it was helpful to have a rehearsed plan or time to reflect on what worked in the past, especially when trying to avoid getting overwhelmed.

“*Obviously, it’s not always easy to realise that whilst you’re in the situation….Time to reflect and realise that you may have not been in the right position or what you need to do because sometimes it does take me quite a while to decide, like my action, what I need to do and I-- maybe who to talk to or where to go and what to do*.” (male, 15, ADHD)

Participants with a dual diagnosis described how learning from previous experiences helped them stay calm and emotionally regulated.

“*Because I used to get angry and went through a lot of these sorts of situations, I can actually manage my anger like I don’t, I can actually, like, manage my anger quite well before the trigger. I don’t really have that short of a fuse. On the contrary to what most people would think.”* (male, 14, dual diagnosis)

Autistic participants reflected on the importance of separating their identity from deficit-based narratives and protecting their confidence.

“*When like, if you see, like, a news article or something, it kind of comes across as-- so if it’s like based on autism, it comes across as something that’s kind of like hurting people? And people display it as something that means you’re incapable and like, you can’t be independent and things like that but it, yeah. […] Kind of starts to destroy my confidence, if like, I need to remember that it’s not who I am.*” (female, 12, autism)

Building a positive self-image was seen as essential in developing self-compassion and taking steps to avoid emotionally triggering situations.

“*Yeah, just where other people are talking and I struggle to find a time when I can start talking because I struggle to know when people have stopped talking, I often just try to avoid conversations so that that doesn’t happen in the first place.”* (male, 12, autism)

“*So that’s why I don’t go for lunch anymore. I just think it’s just easier, like they’re still my friends and I see them at break time. I just think it’s better off like that*.” (female, 15, autism)

## Discussion

This qualitative interview study explored the perspectives of 57 neurodivergent adolescents - diagnosed with autism, ADHD, or both - on how they prepare for and manage their reactions to emotionally upsetting experiences. Our analysis centres on regulation strategies across home and school; complementary findings on the school-based triggers of upsetting experiences are reported elsewhere^26^. This focus is key to efforts to design or adapt interventions to support emotion regulation in neurodivergent youth and aligns with the neurodiversity perspective central to the RE-STAR framework, which emphasises the value of *‘insider’* knowledge and a strengths-based, identity-affirming approach^40^.

Existing literature has often underemphasised the role of environmental and interpersonal supports in promoting emotional wellbeing among neurodivergent young people. Our findings extend current guidance^41,42^ that advocates for inclusive, emotionally safe school environments. Participants identified relational warmth, predictability, and autonomy as key protective factors. This suggests school culture prioritising kindness, respect, authentic connection and the intrinsic valuing of each individual, not over-conformity to neurotypical norms, may help reduce both the frequency and severity of everyday distress in neurodivergent people.

These interview findings are at odds with dominant autism theories (e.g., theory of mind and social motivation theory), which posit that autistic young people have poor interpersonal skills, a limited interest in connecting with others and prefer to be by themselves^43,44^. While some autistic young people in our study valued having personal space to decompress, experiencing a sense of belonging and feeling valued by others, were fundamental for handling difficult situations.

With regards to the ADHD group (with or without autism), these young people thought having a sense of agency and control over their environment was important. They noted that young people with ADHD are more likely to experience school punishment, detentions, and exclusions^45^. School staff may also misinterpret these behaviours^46^ and use more controlling and autonomy-thwarting practices with this group in their efforts to “fix” ADHD behaviour or address mood swings and emotional outbursts. Nevertheless, the accounts from neurodivergent young people highlight the need for neurodiversity-informed approaches that move away from rigid behaviour modification systems. Such practices risk contributing to emotionally based school avoidance (EBSA), highlighting the importance of relational, flexible, and autonomy-supportive environments.

Leveraging personal strengths emerged as an important protective strategy for both ADHD and autistic participants, enabling them to maintain emotional balance and resist deficit-based framings of their differences. Rather than, is often the case, traits such as distractibility or humour being viewed solely in the negative, young people reframed them as coping resources, which helped to redirect attention, reduce distress, and preserve confidence. This reframing reflects a strengths-based and affirming identity stance, positioning difference as a source of resilience rather than deficit^31,47^. Planning ahead and reflecting on past successes also contributed to a sense of competence and mastery, reinforcing young people’s belief in their ability to regulate emotions effectively.

These strategies resonate with the core tenets of self-determination theory, particularly the need for autonomy, competence, and relatedness as drivers of motivation and well-being^48^. For autistic participants in particular, actively resisting stigmatizing discourses and choosing when to withdraw from triggering situations were described as deliberate acts of agency and self-protection. While such behaviours may be misinterpreted by others as avoidance, participants themselves framed them as essential to safeguarding self-worth. Taken together, these findings highlight the importance of recognising neurodivergent adolescents as active agents in shaping their emotional worlds, and of embedding identity-affirming, strengths-based approaches in clinical and educational contexts.

**Fig. 5:**
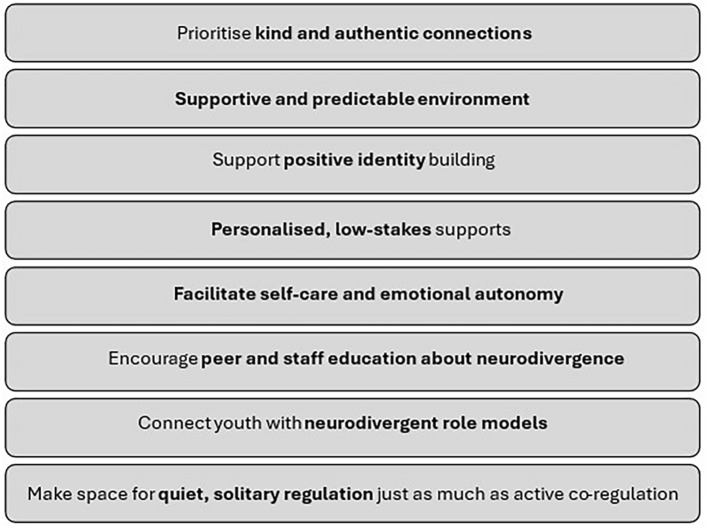
Summary of implications for practice.

Clinical implications from these findings point to the importance of tailoring support so that neurodivergent adolescents are active partners in shaping their own wellbeing. In clinical settings, this means framing therapy around agency, belonging, and collaboration^26,31^, and providing opportunities for adolescents to identify and apply their own strengths. Within schools, implications extend to moving beyond behaviourist approaches toward relational and autonomy-supportive practices, where students are engaged in co-designing strategies that work for them. For families, everyday practices that validate emotions, encourage self-advocacy, and create predictable yet flexible routines can reinforce resilience. By positioning young people as experts in their own experiences, professionals and caregivers can promote not only emotion regulation but also long-term identity development and wellbeing. These practice recommendations directly complement the applied strategies summarised in Fig. 5, illustrating how insights from adolescents themselves can inform practical guidance across home, school, and clinical contexts.

Beyond clinical contexts, the implications for practice are rich and multifaceted. They highlight critical relational, environmental, and personal factors that support neurodivergent young people as they seek to manage everyday challenges^26^ and the emotional burden these evoke in school we previously reported^27^.

Fostering inclusive environments for neurodivergent individuals requires a foundational shift toward prioritizing kindness and authentic interpersonal connection. Rather than emphasizing conformity to neurotypical norms, educators, caregivers, and peers should adopt acceptance-based approaches that affirm individual differences. Small yet meaningful actions - such as using respectful language, acknowledging personal strengths, and responding with empathy - can significantly support emotional regulation and wellbeing. Importantly, findings from the study highlight a clear need for predictability and preparation in daily routines, as unpredictability was frequently associated with distress. Consistent, trusting relationships with adults and peers also play a critical role in promoting stability. Structures that ensure relational continuity, such as consistent support staff or mentorship schemes, can strengthen co-regulation capacities and emotional safety.

Surprisingly, few participants framed their challenges in terms of internal deficits, and most did not refer to standardised emotion regulation strategies suggested by school staff^49^. Instead, external stressors and environments emerged as key contributors to their dysregulation. Overall, affirming neurodivergent identities, adapting environments, and fostering emotional expression offer powerful alternatives to deficit-based frameworks. Neurodivergent-affirming spaces, flexible supports (e.g., sensory-friendly areas, seating choices), and recognition of agency were identified as essential in promoting wellbeing. Allowing young people to choose their self-regulation strategies, while also validating their right to express frustration and distress as a legitimate emotional release, was reported to be critical. Being accepted, valued, and included, not simply accommodated, was a recurring theme to prevent negative emotions. Moreover, while commonalities between ADHD and autism supports were noted, participants also articulated important distinctions in how needs and preferences manifest in interactions. Adolescents with ADHD emphasized the importance of loyalty and reciprocity in friendships and a need to leverage own strengths to get through adversity. In contrast, autistic participants placed greater value on consistency, predictability, and co-regulation, highlighting a preference for calm and stable interactions.

### Strengths and limitations

This study has several strengths, including a large sample of 57 adolescents across three diagnostic groups and a co-designed, interactive interview schedule developed with Y-RP members to elicit richer accounts. Analysis was further refined through ongoing collaboration with the Y-RP. These decisions reflect not only methodological rigour but also a commitment to valuing young people’s own experiences as central to the research process. Compared to other visual research methods such as photovoice, our approach offered greater flexibility by allowing participants to choose creative mediums that felt familiar and comfortable, including drawing, Lego or clay modelling, and photography. This personalised design enabled young people to represent their experiences in ways that matched their own preferences and abilities, thereby reducing barriers to participation and enhancing the authenticity of the data generated. Importantly, the creative tasks also allowed young people to bring their own agenda into the interviews and to initiate discussions that researchers may otherwise have missed.

Limitations include the exclusion of non-speaking neurodivergent adolescents with or without learning disability and those outside mainstream education or in alternative provision, limiting the generalisability of the findings. Additionally, background data on mental health and co-occurring conditions were not systematically collected meaning that no conclusions can be drawn on how these might impact neurodivergent young people’s emotional experiences and responses. Another limitation is the lack of detailed information on participants’ broader school coping, making it difficult to know whether the sample reflects those already managing well or also those facing greater challenges.

Finally, the influence of intersecting identities, such as gender, ethnicity, and socioeconomic status, on emotional experiences warrants further exploration in future research. Future research should also extend these findings by involving neurodivergent adolescents in the co-design of support schemes that explicitly build on their strengths and identity-protective strategies. Co-producing translational science with neurodivergent young people would ensure that supports are not only acceptable and feasible, but also meaningful and sustainable in real-world settings. Longitudinal and context-specific studies (e.g., across home, school, and clinical environments) are also needed to explore how emotion regulation strategies evolve over time, and how structural factors such as school culture and family practices shape these developmental trajectories.

## Conclusion

This study offers valuable insights into the existing approaches that young people with diagnoses of autism, ADHD or both diagnoses employ to prevent or manage negative emotions during upsetting experiences. These accounts provide a new perspective with the potential to shape new interventions that can help support neurodivergent people to better manage the challenges they face in their everyday lives and ultimately help mitigate their wellbeing and life chances.

## Supplementary Information

Supplementary Information.

## Data Availability

Copies of the original codes, and anonymised quotes and transcripts will be available from the corresponding author upon reasonable request.

## References

[1] Lai, M.-C. et al. Prevalence of co-occurring mental health diagnoses in the autism population: a systematic review and meta-analysis. *Lancet Psychiatr.* **6**, 819–829 (2019). 10.1016/S2215-0366(19)30289-5 31447415

[2] Munkhaugen, E. K., Gjevik, E., Pripp, A. H., Sponheim, E. & Diseth, T. H. School refusal behaviour: Are children and adolescents with autism spectrum disorder at a higher risk?. *Res. Autism Spectr. Disord.* **41**, 31–38 (2017).

[3] Thambirajah, M., Grandison, K. J. & De-Hayes, L. *Understanding school refusal: A handbook for professionals in education health and social care* (Jessica Kingsley Publishers, 2008).

[4] National Autistic Soceity. *Education Report 2023*. Report available at https://www.autism.org.uk/what-we-do/news/education-report-2023 (2023).

[5] National Office of Statistics. *Educational experiences of young people with special educational needs and disabilities in England*. Survey report available at https://www.ons.gov.uk/peoplepopulationandcommunity/educationandchildcare/bulletins/educationalexperiencesofyoungpeoplewithspecialeducationalneedsanddisabilitiesinengland/februarytomay2022 (2023).

[6] Connolly, S. E., Constable, H. L. & Mullally, S. L. School distress and the school attendance crisis: a story dominated by neurodivergence and unmet need. *Front. Psychiatr.* **14**, 1237052 (2023). 10.3389/fpsyt.2023.1237052 PMC10556686 37810599

[7] Bodalski, E. A., Knouse, L. E. & Kovalev, D. Adult ADHD, Emotion Dysregulation, and Functional Outcomes: Examining the Role of Emotion Regulation Strategies. *J. Psychopathol. Behav. Assess.* **41**, 81–92. 10.1007/s10862-018-9695-1 (2019).

[8] Eyre, O. et al. Irritability in ADHD: Associations with depression liability. *J. Affect. Disord.* **215**, 281–287 (2017). 28363151 10.1016/j.jad.2017.03.050 PMC5409953

[9] Faraone, S. V. et al. Practitioner Review: Emotional dysregulation in attention-deficit/hyperactivity disorder - implications for clinical recognition and intervention. *J. Child Psychol. Psychiatr.* **60**, 133–150. 10.1111/jcpp.12899 (2019). 10.1111/jcpp.12899 29624671

[10] Seymour, K. E. et al. Emotion regulation mediates the relationship between ADHD and depressive symptoms in youth. *J. Abnorm. Child Psychol.* **40**, 595–606. 10.1007/s10802-011-9593-4 (2012). 22113705 10.1007/s10802-011-9593-4

[11] Shaw, P., Stringaris, A., Nigg, J. & Leibenluft, E. Emotion dysregulation in attention deficit hyperactivity disorder. *Am. J. Psychiatr.* **171**, 276–293. 10.1176/appi.ajp.2013.13070966 (2014). 24480998 10.1176/appi.ajp.2013.13070966 PMC4282137

[12] Sobanski, E. et al. Emotional lability in children and adolescents with attention deficit/hyperactivity disorder (ADHD): clinical correlates and familial prevalence. *J. Child Psychol. Psychiatr.* **51**, 915–923. 10.1111/j.1469-7610.2010.02217.x (2010). 10.1111/j.1469-7610.2010.02217.x 20132417

[13] Cai, R. Y., Richdale, A. L., Uljarević, M., Dissanayake, C. & Samson, A. C. Emotion regulation in autism spectrum disorder: Where we are and where we need to go. *Autism Res.* **11**, 962–978. 10.1002/aur.1968 (2018). 29979494 10.1002/aur.1968

[14] Carter Leno, V. et al. Behavioural and physiological response to frustration in autistic youth: associations with irritability. *J. Neurodev. Disord.* **13**, 27. 10.1186/s11689-021-09374-1 (2021). 34275441 10.1186/s11689-021-09374-1 PMC8287810

[15] Cibralic, S., Kohlhoff, J., Wallace, N., McMahon, C. & Eapen, V. A systematic review of emotion regulation in children with Autism Spectrum Disorder. *Res. Autism Spectr. Disord.* **68**, 101422. 10.1016/j.rasd.2019.101422 (2019).

[16] Conner, C. M. et al. Emotion Dysregulation is Substantially Elevated in Autism Compared to the General Population: Impact on Psychiatric Services. *Autism Res.* **14**, 169–181. 10.1002/aur.2450 (2021). 33815651 10.1002/aur.2450 PMC8018538

[17] Mayes, S. D., Calhoun, S. L., Murray, M. J., Ahuja, M. & Smith, L. A. Anxiety, depression, and irritability in children with autism relative to other neuropsychiatric disorders and typical development. *Res. Autism Spectr. Disord.* **5**, 474–485. 10.1016/j.rasd.2010.06.012 (2011).

[18] Smith, I. C. & White, S. W. Socio-emotional determinants of depressive symptoms in adolescents and adults with autism spectrum disorder: A systematic review. *Autism* **24**, 995–1010 (2020). 32191120 10.1177/1362361320908101

[19] Kusché, C. A., Greenberg, M. T. & Anderson, L. A. *The PATHS curriculum: Promoting alternative thinking strategies* (Developmental Research & Programs Seattle, 1994).

[20] Kuypers, L. The zones of regulation: A framework to foster self-regulation. *Sens. Integr. Special Interest Sect. Q.* **36**, 1–4 (2013).

[21] Conner, C. M. et al. Improving emotion regulation ability in autism: The Emotional Awareness and Skills Enhancement (EASE) program. *Autism* **23**, 1273–1287. 10.1177/1362361318810709 (2019). 30400749 10.1177/1362361318810709

[22] Breaux, R. & Langberg, J. M. Development and refinement of the RELAX intervention, an intervention targeting emotion dysregulation and interpersonal conflict in adolescents with ADHD: Results from a pilot study. *Evid.-Based Pract. Child Adolesc. Ment. Health* **5**, 147–163 (2020).

[23] Bennett, J., Parsons, S. & Kovshoff, H. Developing the emotion regulation skills of autistic pupils in educational settings: A systematic literature review. *Journal of Research in Special Educational Needs* (2024).

[24] Mazefsky, C. A. et al. The role of emotion regulation in autism spectrum disorder. *J. Am. Acad. Child Adolesc. Psychiatr.* **52**, 679–688. 10.1016/j.jaac.2013.05.006 (2013). 10.1016/j.jaac.2013.05.006 PMC3719386 23800481

[25] Sonuga-Barke, E. J. et al. Participatory translational science of neurodivergence: model for attention-deficit/hyperactivity disorder and autism research. *Br. J. Psychiatr.* **224**, 127–131 (2024). 10.1192/bjp.2023.151 PMC10933558 38362636

[26] Pavlopoulou, G. *et al.* Upsetting experiences in the lives of neurodivergent young people: A qualitative analysis of accounts of adolescents diagnosed with attention‐deficit/hyperactivity disorder and/or autism. *JCPP Advances*, e70038 (2025). 10.1002/jcv2.70038 PMC13260670 42291673

[27] Lukito, S. *et al.* Emotional burden in school as a source of mental health problems associated with ADHD and/or autism: Development and validation of a new co‐produced self‐report measure. *Journal of Child Psychology and Psychiatry* (2025). 10.1111/jcpp.70003 PMC12447693 40707015

[28] Ferreira, R. D. S. & Castro, T. H. C. D. Participatory and Inclusive Design Models from the Perspective of Universal Design for Children with Autism: A Systematic Review. *Educ. Sci.* **14**, 613 (2024).

[29] Kakoulidou, M. et al. Deepening the participation of neurodivergent youth in qualitative mental health research: Co-development of a general approach and the evaluation of its implementation in a study on emotion. *JCPP Adv.* **4**, e12287 (2024). 39734931 10.1002/jcv2.12287 PMC11669790

[30] National Institute for Health Research. *Payment guidance for researchers and professionals*. https://www.nihr.ac.uk/payment-guidance-researchers-and-professionals (2024).

[31] McGreevy, E. *et al.* An experience sensitive approach to care with and for autistic children and young people in clinical services. *Journal of Humanistic Psychology*, 00221678241232442 (2024).

[32] Pavlopoulou, G. A good night’s sleep: Learning about sleep from autistic adolescents’ personal accounts. *Front. Psychol.* **11**, 583868 (2021). 10.3389/fpsyg.2020.583868 PMC7814098 33469436

[33] Catalani, C. & Minkler, M. Photovoice: A review of the literature in health and public health. *Health Educ. Behave.* **37**, 424–451 (2010). 10.1177/1090198109342084 19797541

[34] Pavlopoulou, G., Burns, C., Cleghorn, R., Skyrla, T. & Avnon, J. I often have to explain to school staff what she needs. School experiences of non-autistic siblings growing up with an autistic brother or sister. *Res. Dev. Disab.* **129**, 104323 (2022). 10.1016/j.ridd.2022.104323 35988460

[35] Pearson, A. *et al.* “I guess when a lot of people outwardly don’t like you, you start to find a dislike within yourself”: Experiences of belonging among autistic adolescents assigned female at birth in mainstream school settings. *School Mental Health* (2025).

[36] Braun, V. & Clarke, V. Using thematic analysis in psychology. *Qual. Res. Psychol.* **3**, 77–101 (2006).

[37] Braun, V. & Clarke, V. Reflecting on reflexive thematic analysis. *Qual. Res. Sport, Exerc. Health* **11**, 589–597 (2019).

[38] Campbell, K. A. et al. Reflexive thematic analysis for applied qualitative health research. *Qual. Rep.* **26**, 2011–2028 (2021).

[39] Morse, J. M., Barrett, M., Mayan, M., Olson, K. & Spiers, J. Verification strategies for establishing reliability and validity in qualitative research. *Int. J. Qual. Methods* **1**, 13–22 (2002).

[40] Sonuga-Barke, E. J. Paradigm ‘flipping’to reinvigorate translational science: Outlining a neurodevelopmental science framework from a ‘neurodiversity’perspective. *J. Child Psychol. Psychiatr.* **64**, 1405–1408 (2023). 10.1111/jcpp.13886 37706585

[41] Department for Education. *Promoting and supporting mental health and wellbeing in schools and colleges*. Report available at https://www.gov.uk/guidance/mental-health-and-wellbeing-support-in-schools-and-colleges (2021).

[42] National Institute for Health and Care Excellence. *Guidline: Social, emotional and mental wellbeing in primary and secondary education*. https://www.nice.org.uk/guidance/ng223/documents/draft-guideline (2022) 36787393

[43] Baron-Cohen, S. *Mindblindness: An essay on autism and theory of mind* (MIT press, 1997).

[44] Chevallier, C., Kohls, G., Troiani, V., Brodkin, E. S. & Schultz, R. T. The social motivation theory of autism. *Trends Cogn. Sci.* **16**, 231–239 (2012). 22425667 10.1016/j.tics.2012.02.007 PMC3329932

[45] John, A. et al. Association of school absence and exclusion with recorded neurodevelopmental disorders, mental disorders, or self-harm: a nationwide, retrospective, electronic cohort study of children and young people in Wales UK. *Lancet Psychiatr.* **9**, 23–34 (2022). 10.1016/S2215-0366(21)00367-9 PMC8674147 34826393

[46] Rogers, M. & Tannock, R. Are classrooms meeting the basic psychological needs of children with ADHD symptoms? A self-determination theory perspective. *J. Attention Disord.* **22**, 1354–1360 (2018). 10.1177/1087054713508926 24327276

[47] Botha, M. & Gillespie-Lynch, K. Come as you are: Examining autistic identity development and the neurodiversity movement through an intersectional lens. *Human Dev.* **66**, 93–112 (2022).

[48] Deci, E. L., Olafsen, A. H. & Ryan, R. M. Self-determination theory in work organizations: The state of a science. *Ann. Rev. Organ. Psychol. Organ. Behave.* **4**, 19–43 (2017).

[49] Angus, G. & Nelson, R. B. School-wide positive behavior interventions and supports and student academic achievement. *Contemp. School Psychol.* **25**, 443–465 (2021).

