# Supplementary material for: Situating emotion regulation in autism and ADHD through neurodivergent adolescents’ perspectives
Source: Sci Rep. 2025 Oct 27;15:37464. doi: 10.1038/s41598-025-21208-x (PMC12559169; doi:10.1038/s41598-025-21208-x)
Supplement: Supplementary file 1 — Supplementary Information. [file 41598_2025_21208_MOESM1_ESM.docx]

**SUPPLEMENATRY MATERIALS**

**Supplementary material I: Creative task prompts**

Participants received the following information on how to create something to describe their emotions (excerpt from participant information sheet below):

**Supplementary material II: Examples of creative tasks completed by participants**


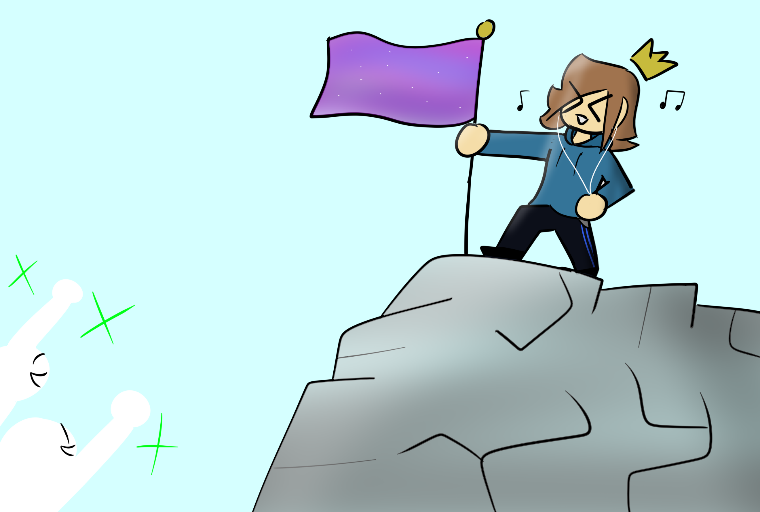


Figure 1. An illustration showing the young person feeling confident when listening to music (Male, 15, dual diagnosis)


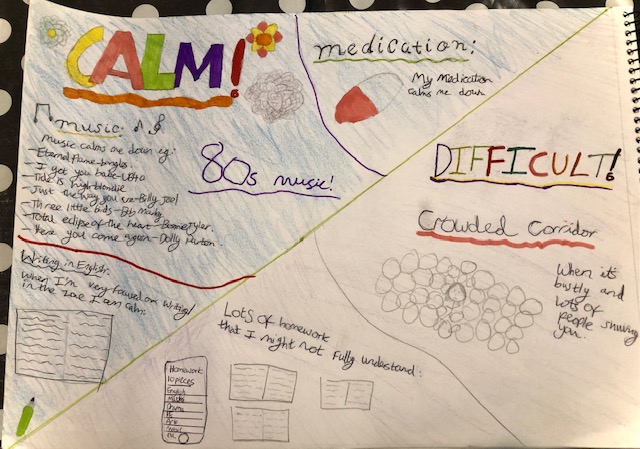


Figure 2. A drawing showing how listening to music and taking a medication can help with negative emotions (Female, 12, ADHD)

**
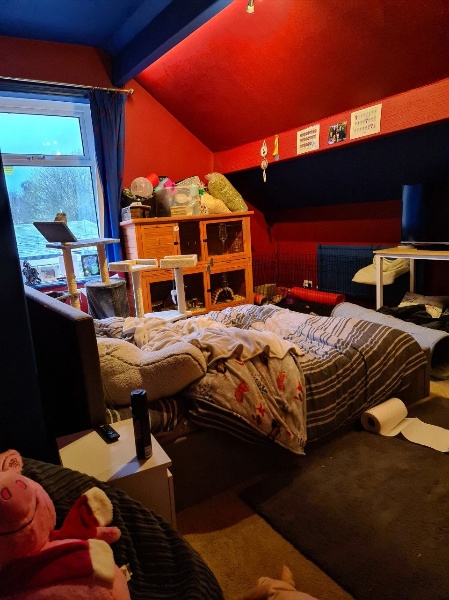
**

Figure 3. “That first one (photo) is my room where I'm calm because it's just my room. So I can be alone and relax there.” (Male, 12, autism)


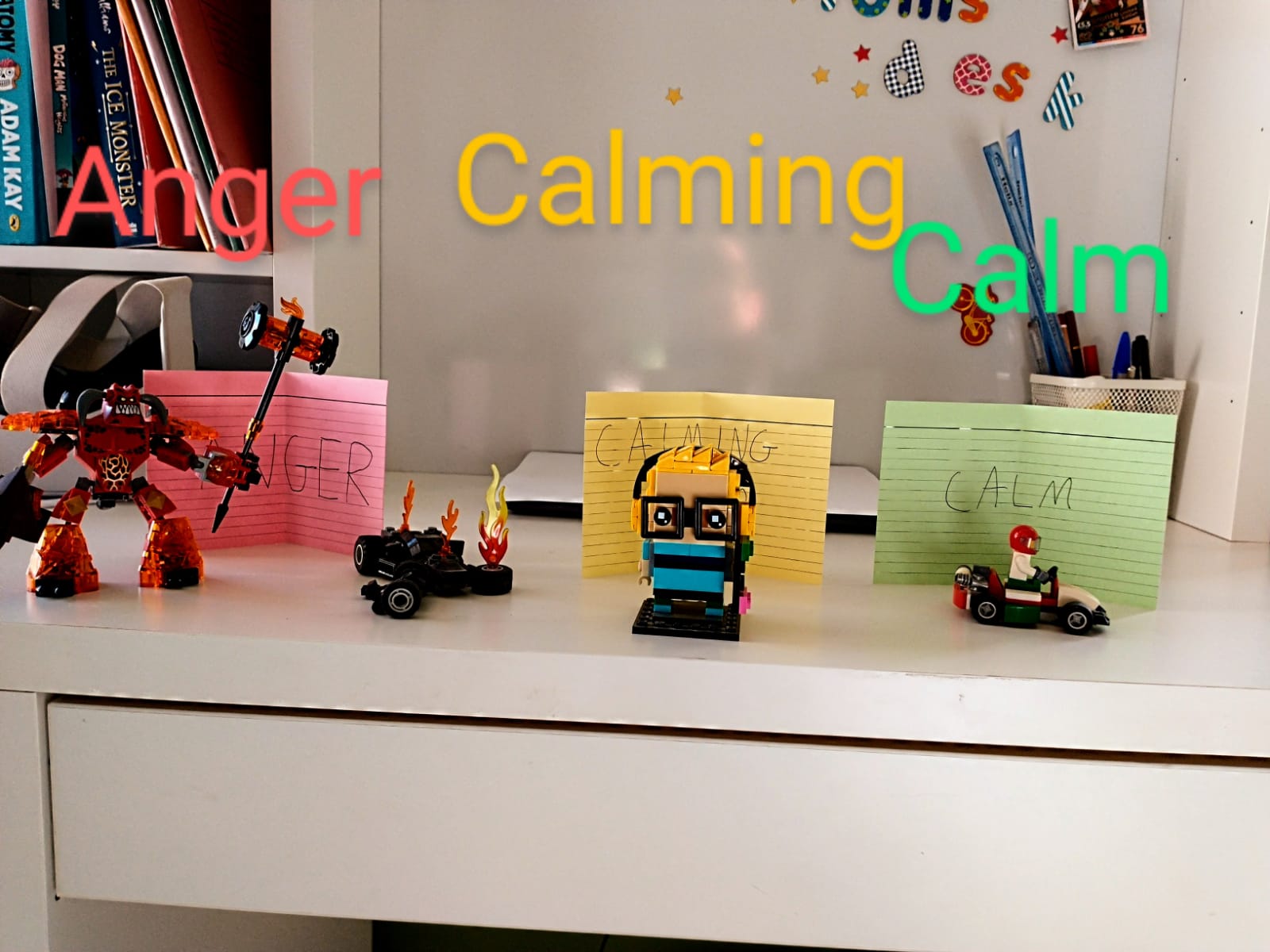


Figure 4. “Well, this one is when I'm calming down, but not completely calm. I've just used my -- this one I actually didn't do anything extra to, because this one was already completely built. But, it's got headphones on because I listen to music sometimes to calm down. It's got a Nintendo Switch under his arm as I sometimes play games when I'm calming down. And that's really it.” (Male, 12, dual diagnosis)


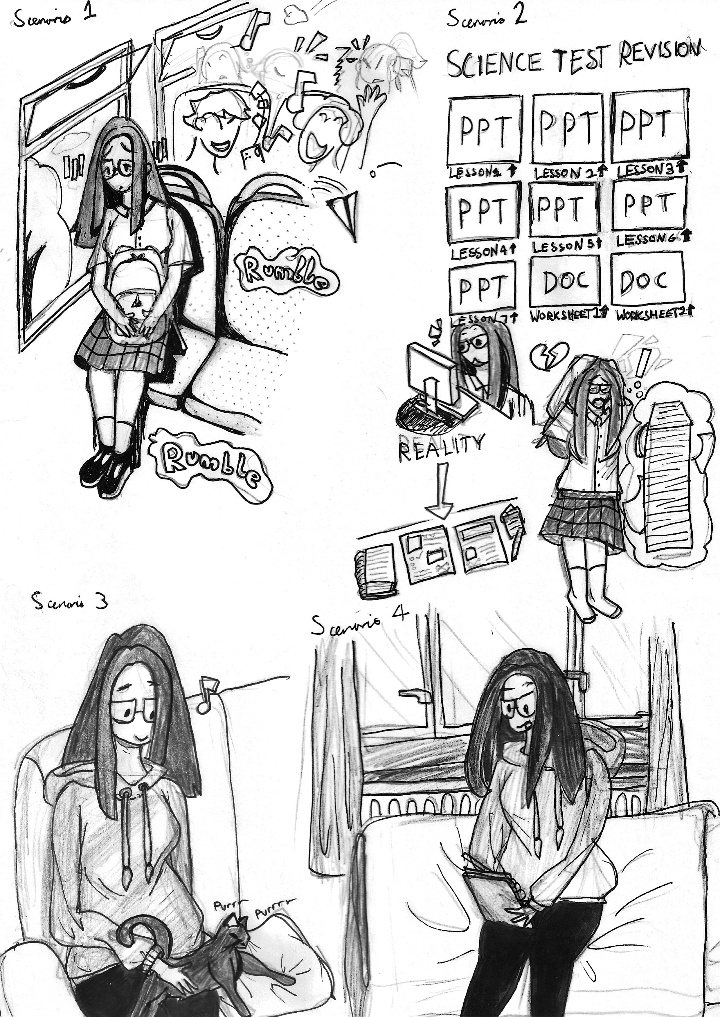


Figure 5. “Well, it's me petting my cat …He really likes cuddles. So, like, I'll normally go on the sofa or my bed or something, he'll come up to me and I'll stroke him for like, wow, sometimes even like an hour or something. And it's just a really big stress reliever.” (Female, 13, autism)

**Supplementary material III: Interview prompts**

1. **ICE-BREAKERS**
2. **Can you tell me how old you are?**
3. **What are your hobbies/interests?**
4. **Can you describe what a typical day is like for you?** *[prompts for morning/school/after school/evening].*
5. **MY EMOTIONS AND ME: CREATIVE TASK**

**Description:** In this task, the participant will present an art/craftwork that we have assigned for them to do at home before the session. We will discuss with the young people how this art/craftwork represents their emotions.

**Instruction:** *Now, we’ve asked you to make something to express the times that are calming, reassuring or difficult and upsetting or both, and reflect on your reactions. Shall we look at what you’ve made?*

1. ***What is it?***
   1. *Please read/show/describe to me what you’ve written/made.*
   2. *Does it have a name/title (e.g., X)?*
2. ***How does this (or X) relate to you being feeling this emotion?***
   1. *What are the situations that X remind you of…?*
   2. *What kind of emotion do you feel then?*
   3. *What makes it emotional for you? How is it different from the usual emotion you have?*
3. ***How long do you feel that way?***
   1. *Did it keep going? What kept it going?*
   2. *Did the feeling change over time?*
4. ***How do these feelings stop?***
   1. *Do they go away on their own?*
   2. *Do you have to do anything to handle them? What are they?*
5. ***What else can you tell me about X?***
6. **MY EMOTIONS AND ME: RESPONDING TO SCENARIOS**

**Description:** Participants will be asked to select a vignette from a selection (of up to 8) presented on their screen. These will be displayed as coloured cards on the screen with a short title. Potential vignette topics are listed below. When the young person clicks on a card, a short video clip will play (for 20-30 secs). In the clip, a young person with a diagnosis of ADHD and/or autism, will appear and briefly narrate a scenario. This will include captions. At the end of the video, a photo or drawing will appear inviting the participant to consider whether this situation is relevant to their own lives, and how this (or a similar situation) would affect them. We will stay curious about what it is like for the young person, and explore what the internal and external factors are in each situation.

We will then go through the questions below with the young person.

**Example vignette**

In school, we worked in pairs and each person had to write a summary of a text. On the day of the presentation, my partner hadn't done his part and blamed me, saying I didn't give him the correct information. (photo of a school classroom)

**Instruction**: *Ok, I will now ask you to look at different scenarios and think: whether you have ever been in similar situations; what your emotional reactions were; and how you managed (or didn’t) to stop these emotions. Let’s start with you choosing which scenario we would like us to start with by clicking on the box of your preference. Once you click on the box, a short clip will play presenting you with a scenario.* *I will ask you 5 questions for each card. Ready? (showing card/slide/vignette). There are no right or wrong answers.*

1. ***Is that situation familiar to you?***
   1. Have you ever been in a situation where …
2. ***What's your emotional reaction in a similar situation?***
   1. *How do you feel then? You can use the emotion chart to help describe your feeling.*
3. ***How do you express your emotion in that situation?***
4. ***What is your reaction to other people when you feel that way?***
5. ***What is the reaction of others to you when you feel that way?***
6. ***Have you ever managed not to feel that way?***
   1. *Could you change your feelings once they have started? What had helped you to stop the feelings before they started?*
   2. *Did your environment allow you or stop you to do what you needed to stop these feelings?*
7. ***How was it after you had experienced the feelings?***
   1. *For example, some young people have told us they couldn’t get to sleep that night thinking about this. What about you? What do you do after a similar situation? What would you do?*

**D. MY EMOTIONS AND ME: THINKING ABOUT DIFFERENT EMOTIONS**

**Description:** In this segment, we will show a colour-coded chart containing words (and/or emojis) that describe different clusters of emotions (see below). We will ask the young person to pick one emotion from each colour and go through the questions below. If appropriate, we could consider a participatory approach, e.g., by presenting the task in a playful manner, to help the young person access the interview. For instance, we can ask the young people to decorate the emoji or to match the emoji with a colour zone/emotion. We will make no prior assumption of what the chosen emotion means. Rather, we will stay open to the young person’s definition. We will stay curious as to what it is like for the young person and explore what the internal and external factors are, i.e., check if the person is able to recognise traits in themselves and others/environment.

**GREEN: Calm, Happy, Focused, Ready to Learn/ Play/Interact with others, Content, Excited**

**BLUE: Sad, Bored, Tired, Sick, Disappointed**

**YELLOW: Worried, Silly, Frustrated, Stressed, Hurt, In pain**

**RED: Mad, Upset/Loud, Scared, Angry, Out of Control, Irritable**


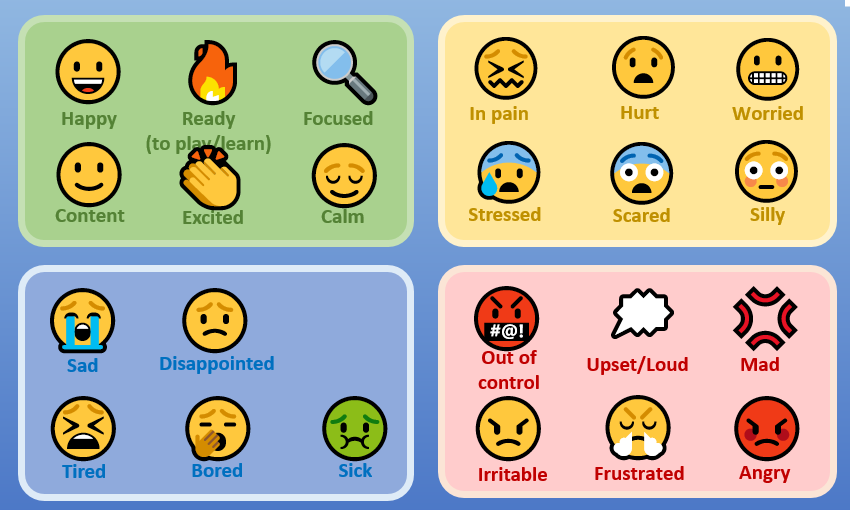


**Instruction:** *I will show you some words that express different emotions. I will ask you what situations bring these feelings at home, school or elsewhere, how long these feelings last, how you handle them and how you stop them. Let’s pick a colour.*

1. ***So, what was it in your life at home or school that has made you feel X?***
   1. *Or can you tell me about a time at home or school when you felt X?*
2. ***Are there times/occasions that you are more likely to feel X? Is it at home or school? Is it during or after an event?***

**Useful additional general prompts (see the above for examples of when to ask the prompts):**

- *How is this related to how you experience X?*
- *How does that make you feel?*
- *What do you do when you feel like this?*
- *Can you tell me about a time when…?*
- *Reflect by repeating a word and wait to hear clarification.*
- *Can you go back and tell me a little bit more about a time when…*
- *What do you mean by “bad”? Help me understand what “bad” looks like for you.*

***Is there anything that I haven’t asked you about X that you’d like to tell me?***
